# Heterologous expression of *Gaeumannomyces graminis* lipoxygenase in *Aspergillus nidulans*

**DOI:** 10.1186/s13568-014-0065-4

**Published:** 2014-08-21

**Authors:** Ruud Heshof, J Paul van Schayck, Juan Antonio Tamayo-Ramos, Leo H de Graaff

**Affiliations:** 1 Laboratory of Systems and Synthetic Biology, Microbial Systems and Synthetic Biology, Dreijenplein 10, Wageningen 6703 HB, Netherlands

**Keywords:** Aspergillus, Lipoxygenase, Gaeumannomyces graminis, Protease, Proteomics, Thioredoxin reductase

## Abstract

*Aspergillus* sp. contain *ppo* genes coding for Ppo enzymes that produce oxylipins from polyunsaturated fatty acids. These oxylipins function as signal molecules in sporulation and influence the asexual to sexual ratio of *Aspergillus* sp. Fungi like *Aspergillus nidulans* and *Aspergillus niger* contain just *ppo* genes where the human pathogenic *Aspergillus flavus* and *Aspergillus fumigatus* contain *ppo* genes as well as lipoxygenases. Lipoxygenases catalyze the synthesis of oxylipins and are hypothesized to be involved in quorum-sensing abilities and invading plant tissue. In this study we used *A. nidulans* WG505 as an expression host to heterologously express *Gaeumannomyces graminis* lipoxygenase. The presence of the recombinant LOX induced phenotypic changes in *A. nidulans* transformants. Also, a proteomic analysis of an *A. nidulans* LOX producing strain indicated that the heterologous protein was degraded before its glycosylation in the secretory pathway. We observed that the presence of LOX induced the specific production of aminopeptidase Y that possibly degrades the *G. graminis* lipoxygenase intercellularly. Also the presence of the protein thioredoxin reductase suggests that the *G. graminis* lipoxygenase is actively repressed in *A. nidulans.*

## Introduction

**Figure 1:**
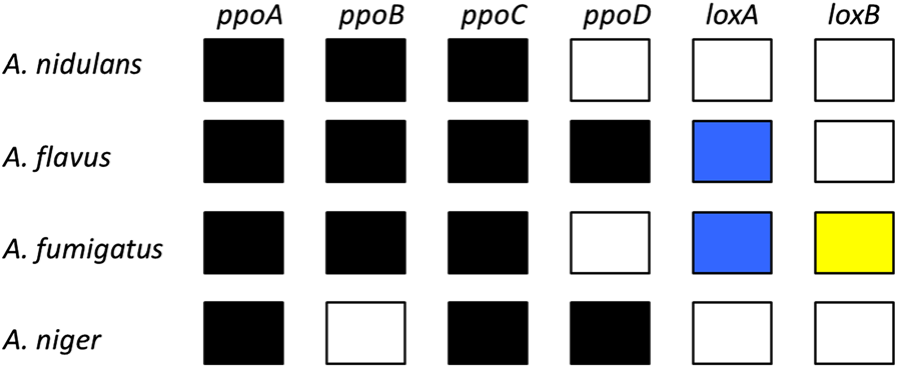
**Distribution of*ppo*and*lox*genes amongst four different*Aspergillus*species.** The black box indicates the presence of the *ppo* gene. The blue box indicates the presence of a *lox* gene coding for an intracellular LOX, and the yellow box represents a *lox* gene coding for an extracellular LOX. *A. nidulans* and *A. niger* have three different *ppo* genes. *A. flavus* has four different *ppo* genes and a *lox* gene. *A. fumigatus* has three different *ppo* genes and two *lox* genes.

*Aspergillus* sp. contains *ppo* genes coding for dioxygenases that belong to the linoleate diol synthase (LDS) protein family. These dioxygenases produce oxylipins called precocious sexual inducer (psi) factors, which is a collective term for C18:1, C18:2 and C18:3 derived oxylipins (Gao et al. [2007]). Oxylipins are signal molecules that are used by fungi to control sporulation (Brodhun and Feussner [2011]). These oxylipins are categorized in three groups depending on the position of hydroxyl groups on the polyunsaturated fatty acid (PUFA): psiB (8′-hydroxy-PUFA), psiC (5′,8′-dihydroxy-PUFA), and psiA, which has a δ-lactone ring at the 5′ position of the psiC oxylipin (Tsitsigiannis et al. [2004]). Depending on the PUFA it is further categorized as α (18:2, linoleic acid), β (18:1, oleic acid), and γ (18:3, linolenic acid) (Tsitsigiannis and Keller [2007]). *Aspergillus nidulans* contains three *ppo* genes: *ppoA*, *ppoB*, and *ppoC* coding for enzymes that synthesize the oxylipins psiBα, psiBβ, and psiBβ respectively (Brodhun and Feussner [2011]). *Aspergillus niger* also contains three *ppo* genes where it lacks *ppoB* but instead contains *ppoD* (Wadman et al. [2009]) In *A. nidulans* PpoA and PpoC have antagonistic roles and PpoB upregulates *ppoA* and represses *ppoC* (Tsitsigiannis et al. [2005]; Brodhun and Feussner [2011]). The deletion of the *ppoA* and *ppoB* genes increase the ratio of asexual to sexual sporulation, while deletion of the *ppoC* gene decreases the ratio of asexual to sexual sporulation. However, it is speculated that these sporulation phenotypes cannot be explained by the psiB oxylipin levels alone (Tsitsigiannis et al. [2005]). Therefore it is suggested that other oxylipins, produced by either Ppo’s or other enzymes, are involved in these phenotypic differences. Lipoxygenase (LOX) is a non-heme iron- or managese-containing dioxygenase present in a wide variety of organisms, including fungi (Heshof et al. [2013]). They catalyze the synthesis of oxylipins, but are absent in *A. nidulans* and *A. niger* (Wadman et al. [2009]). However, the human pathogenic *Aspergillus flavus* and *Aspergillus fumigatus* contain both *ppo* genes and *lox* genes (Brown et al. [2008]; Affeldt et al. [2012]). The distribution of *ppo* and *lox* genes is schematically given in Figure 1. Studies revealed that LOX in *A. flavus* is involved in quorum-sensing and phenotypic differences are seen when the gene is disrupted (Brown et al. [2008]; Affeldt et al. [2012]). The *Gaeumannomyces graminis* LOX is a secreted enzyme that is capable of producing 11*S*-HPODE and 13*R*-HPODE oxylipins and is hypothesized to be involved in invading plant tissue (Oliw [2002]). Plant oxylipins 9*S-*HPODE and 13*S*-HPODE induce sporogenic effects in *A. nidulans* similar to the ones produced by the psi factor (Calvo et al. [1999]). Both oxylipins caused decreased mycelial growth where 13*S*-HPODE in a 10–100 μM concentration also reduces mycotoxin production of aflatoxin and sterigmatocystin (Burow et al. [1997]). In this study we introduced the *G. graminis* LOX in the *A. nidulans* WG505 production host. The goal of the study was to verify whether *A. nidulans* is a suitable host for the heterologous expression of *G. graminis* LOX.

## Materials and methods

### Expression of *G. graminis* LOX in *A. nidulans*

Heterologous expression of *G. graminis* LOX in *A. nidulans* was performed according to previous studies (Nyyssölä et al. [2012]). The gene encoding the *G. graminis* LOX AAK81882.1 was codon-optimized for expression in *A. niger* and synthesized by DNA 2.0 (Menlo Park, USA) [GenBank KM248327]. For expression the promoter and secretion signal of the *xlnD* gene from *A. niger* [GI:74626559] replaced the native secretion signal of *G. graminis* (Van Peij et al. [1997], Van der Straat et al. [2014]). With help of the *Xba*I and *Bam*HI restriction sites the synthesized gene was incorporated into a pUC19 vector and was used to transform *A. nidulans* WG505, that is a *pyrA* derivative of *A. nidulans* WG 096 (ATTC 48756) (Nyyssölä et al. [2012]). The transformants were plated on MMS plates and incubated for 4 days at 37°C (Kusters-van Someren et al. [1991]). Transformants were analyzed by PCR for verification of integration of the *lox* gene into the genome. *A. nidulans* was grown for 48 h at 37°C at 250 rpm in 100 ml MM + 50 mM D-xylose using 500 ml Erlenmeyer flasks. The culture broth was separated from the mycelium by funnel filtration and both were submerged into liquid nitrogen to freeze and preserve the materials. The culture broth and the mycelium were stored at −80°C until further research.

### Growth and induction of *A. nidulans* wild type and transformant

*A. nidulans* WG505 and an isogenic transformant expressing the *G. graminis* LOX (*A. nidulans* GG-LOX) were cultivated on agar plates containing complete medium (6.0 g/l NaNO_3_, 1.5 g/l KH_2_PO_4_, 0.5 g/l KCl, 0.5 MgSO_4_ · 7 H_2_O, 2 g/l peptone, 1 g/l yeast extract, 1 g/l casamino acids, 0.3 g/l yeast ribonucleic acids, 2 ml/l vitamin solution, 1 ml/l Vishniac solution, 50 mM D-(+)xylose, 12% agar) (Pontecorvo et al. [1953]). Also, *A. nidulans* was grown at 37°C in 500 ml Erlenmeyers containing 100 ml complete medium without agar and inoculated with 10^6^ spores/ml. Both media contained 50 mM D-xylose to induce the *xlnD* promotor and stimulate production of *G. graminis* LOX.

### Fermentation of *A. nidulans*

*A. nidulans* WG505 and *A. nidulans* GG-LOX were fermented using a New Brunswick BioFlo® 310 (Eppendorf, Nijmegen, The Netherlands). The batch-phase was performed using 3 l of medium for 24 h (5 g/kg glucose, 0.5 g/kg KH_2_PO_4_, 0.5 g/kg MgSO_4_^.^ 7 H_2_O, 4.0 g/kg (NH_4_)_2_SO_4_, 1 g/kg yeast extract, 0.1 g/kg Struktol J673). When the glucose was completely consumed the fermentation was fed 2 l using D-xylose medium at a speed of 0.35 g/min (75 g/kg D-xylose, 3.1 g/kg KH_2_PO_4_, 14.85 g/kg (NH_4_)_2_SO_4_, 24.4 g/kg yeast extract). Samples were taken every 2 h to test for the presence of the *G. graminis* LOX.

### mRNA isolation and identification of the *G. graminis* LOX

Mycelium from *A. nidulans* WG505 and *A. nidulans* GG-LOX was submerged in peqGOLD TriFast (peqLAB, De Meern, The Netherlands) and disrupted using glass beads and a MP FastPrep-24 beadbeater (MP Biomedicals, Eindhoven, The Netherlands). The RNA isolated was treated with DNase I and transcribed to cDNA using Omniscript RT enzyme (Qiagen, Venlo, The Netherlands). The resulting cDNA was submitted to PCR using the forward primer 5′-TGAGTTGCAGAACTGGATCG-3′ and reverse primer 5′-GCAGAACGCCAGAAAACTTC-3′ for detection of the *G. graminis lox* mRNA. cDNA from positive reactions were sequenced (Baseclear, Leiden, The Netherlands). As a positive control the pyruvate kinase (*pkiA)* gene was amplified using the forward 5′-GCCAGTCTTGAACTGAACGC-3′ primer and the reverse 5′-GCCAGATCTTGACGTTGAAGTC-3′ primer (de Graaff et al. [1992]). Amplified gDNA results in a 304 bp fragment while amplified cDNA results in a 204 bp fragment due to the existence of an intron in the fragment.

### Mutagenesis of the *G. graminis* LOX

To test whether the activity of the *G. graminis* LOX interferes with the correct synthesis and secretion of the protein a double site-directed H306Q-H310E mutagenesis was performed resulting in inactive *A. nidulans* GG-LOX mutants. These mutations do not affect protein expression levels but result in a catalytically inactive enzyme (Cristea et al. [2005]). For mutagenesis the QuikChange Lightning Site-Directed Mutagenesis Kit (Agilent Technologies, Amstelveen, The Netherlands) was used. The forward 5′-GTTCTACTCCCAAATGTACCAGGTGCTGTTCGAGACCATCCCGGAG-3′ primer and the reverse 5′-CTCCGGGATGGTCTCGAACAGCACCTGGTACATTTGGGAGTAGAAC-3′ primer were designed as advised by the protocol of the mutagenesis kit. Positive transformants were identified by sequencing (Baseclear, Leiden, The Netherlands).

### Western blot analysis

To identify the presence of *G. graminis* LOX a western blot analysis was performed. 20 μl of culture broth and disrupted mycelium was isolated and run on a 10% SDS-PAGE with a voltage of 100 V for 1 h using Tris-HEPES-SDS Running Buffer (Thermo Scientific PI28398). Afterwards the gel was rinsed with dH_2_O and pre-soaked with CAPS-blot buffer. A nitrocellulose membrane was used to absorb the proteins from the SDS-PAGE and was blotted overnight at 70 mA. The membrane was washed for 30 min in TBST and afterwards it was blocked using TBST + 1% BSA for 30 min. Rabbit polyclonal antibody of *G. graminis* (Eurogentec, Seraing, Belgium) was used in a 1/1000 dilution to detect LOX on the membrane. After 3x washes with TBST for 10 min the membrane was incubated for 30 min using a 1/1000 dilution of secondary anti-rabbit peroxidase antibody (Sigma-Aldrich Lot. A0545-1ML). After the final 3 × 10 min wash step with TBST the membrane was submitted to AP-detection. This was done by mixing two solvents: 60 mg of 4-chloro-1-naphtol to 20 ml methanol (A) and by adding 60 μl of 30% ice-cold H_2_O_2_ to 100 ml TBS (B). Prior to use solvents A and B were mixed and the membrane was added to this mixture. The reaction was stopped with dH_2_O after 30 min of incubation.

### Immunoprecipitation using *G. graminis* LOX antibody and proteomics analysis

*A. nidulans* WG505 and *A. nidulans* GG-LOX were grown with 50 mM D-xylose for 48 h and the culture broth and mycelium were separated by funnel filtration The mycelium was disrupted three times by French Press with a pressure of 1,000 psi and the resulting cell free extract was prepared by centrifugation. Samples of 5 ml from both culture broth and cell free extract were taken and incubated with 5 ml of polyclonal *G. graminis* antibody (Eurogentec, Seraing, Belgium). The samples were incubated overnight with a stirring speed of 200 rpm at 4°C. Immunoprecipitation was performed using 100 μl Dynabeads Protein G Magnetic Beads (Life Technology, Bleiswijk, The Netherlands). After immunoprecipitation the proteins were separated on a 10% SDS-PAGE at 100 V. The proteins were cut from the SDS-PAGE and submitted to in-gel digestion with 100 ng trypsin/sample in 50 mM ammonium bicarbonate buffer. Afterwards the samples were diluted 1:1 using 2% trifluoroacetic acid to acidify the proteins. The samples were purified by binding the proteins to a reversed-phase C18 column and washing with 0.1% formic acid. Then the proteins were eluted with 80% acetonitrile + 0.1% formic acid. Finally the samples were analyzed by LC-MS/MS in the Radboud Proteomics Centre (Radboud University, Nijmegen, The Netherlands). The resulting data were analyzed using MaxQuant software (Cox and Mann [2008]).

## Results

### Phenotype differences in *A. nidulans*

**Figure 2:**
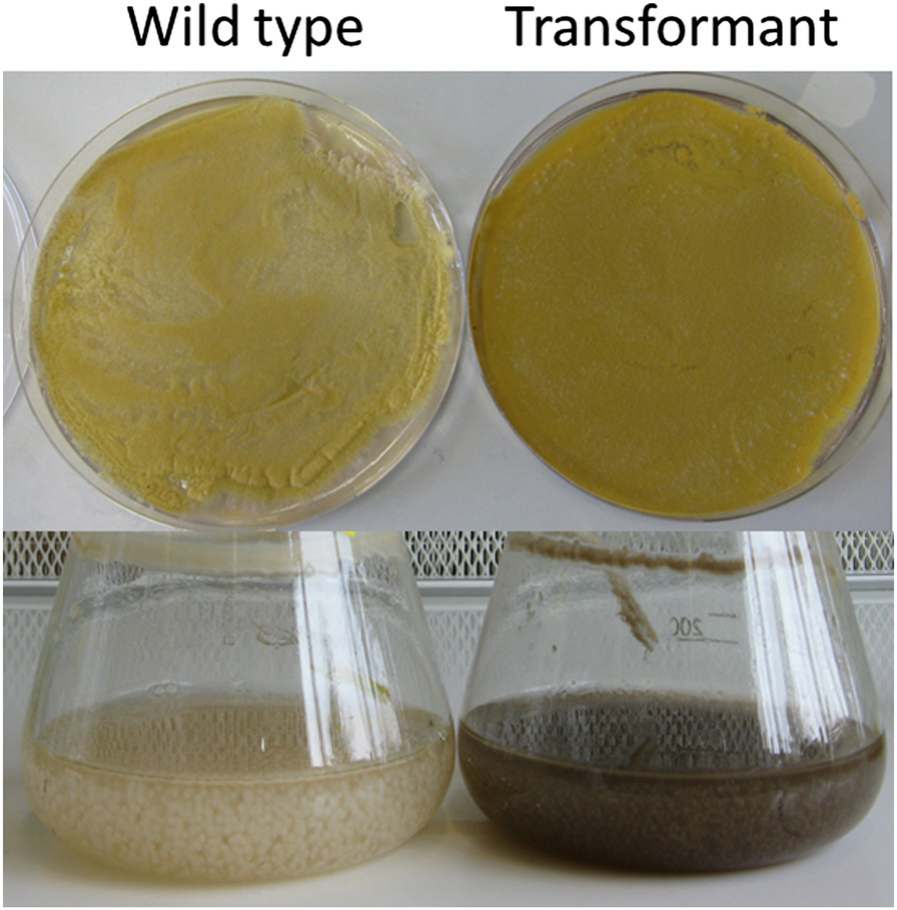
***A. nidulans*wild type and transformant on plates and liquid media after 48 h of growth.** All cultures were induced with 50 mM D-xylose. Phenotypic changes are found, the transformant expressing *G. graminis* LOX becomes brown while the wild type does not.

After 48 h of growth in liquid cultures and on agar plates *A. nidulans* GG-LOX showed a different phenotype from the one of the wild type. As shown in Figure 2, the mycelium of *A. nidulans* GG-LOX showed a browner colour when compared to *A. nidulans* WG505. Also, the pellet size of the transformant was smaller than the one of the wild type strain. The amount of spores formed was also increased in the LOX expressing strain compared to the wild type, both in liquid culture and in agar plates, which could be caused by the produced oxylipins of the *G. graminis* LOX (data not shown).

### mRNA identification of the *G. graminis lox* gene

In order to confirm that the *G. graminis* LOX gene was successfully transcribed in the *A. nidulans* GG-LOX transformant mRNA was isolated from both the wild type and the *A. nidulans* GG-LOX. The analysis was also performed in the wild type strain that was used as a negative control. The mRNA sequence of the transformant resulted in 100% homology with the *G. graminis lox* gene. This implies the gene was correctly transcribed and the production of the *G. graminis* LOX was stopped at another stage during protein synthesis.

### Western blot analysis

**Figure 3:**
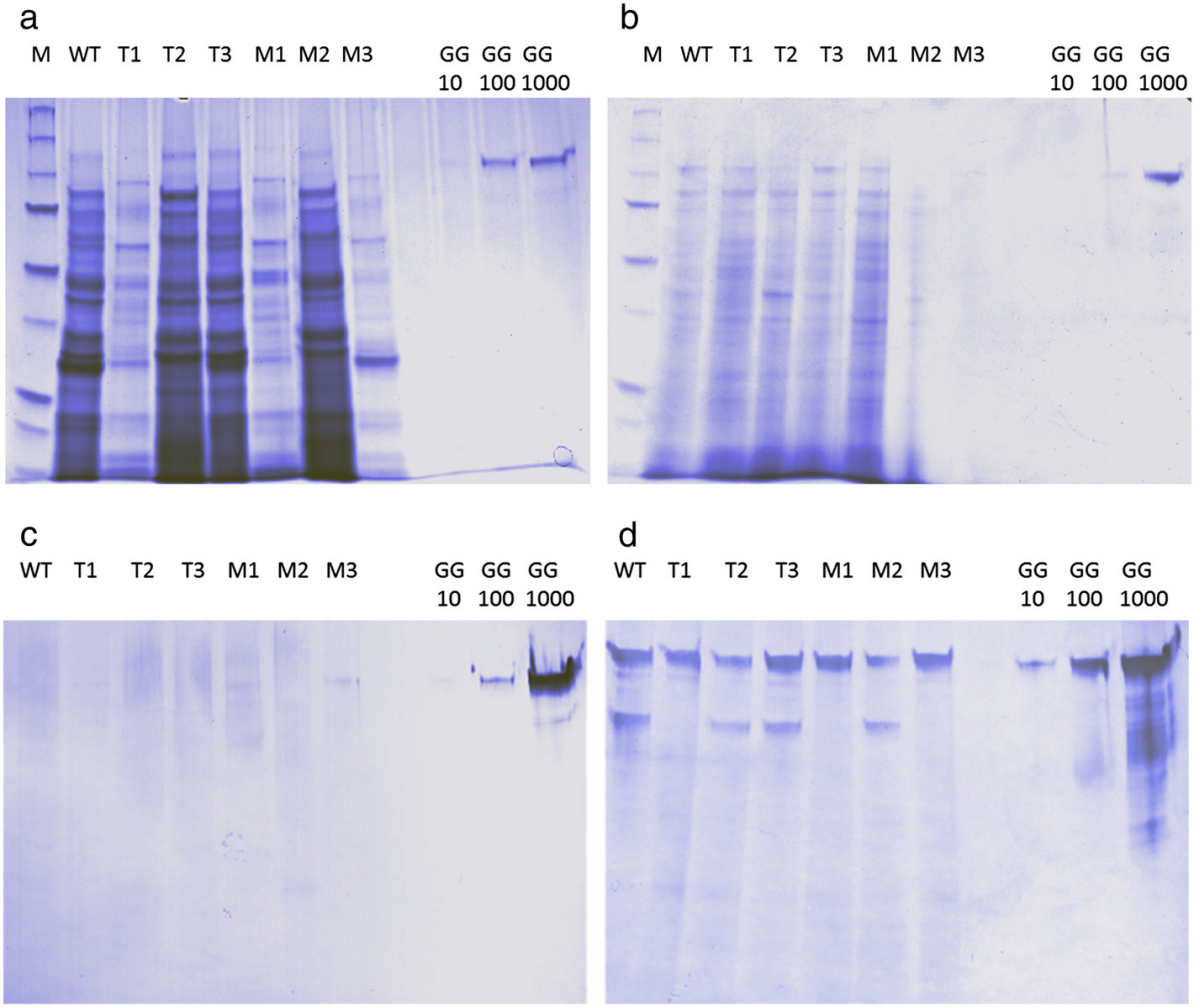
**SDS-PAGE and western blot analysis of*G. graminis*LOX expression*A. nidulans*.** Samples T1-T3 are transformants of *A. nidulans* carrying the *G. graminis lox* gene, while M1-M3 are transformants of *A. nidulans* carrying the mutated *G. graminis lox* gene. As positive controls 10 ng, 100 ng, and 1000 ng of the *G. graminis* LOX were applied. The wild type (WT) sample is used as a negative control. **a)** SDS-PAGE of the proteins in the culture broth of *A. nidulans*; **b)** SDS-PAGE of intracellular proteins of *A. nidulans*; **c)** Western blot of the proteins in the culture broth reacting to the antibodies of the *G. graminis* LOX; **d)** Western blot of intracellular proteins reacting to the antibodies of *G. graminis* LOX.

A western blot analysis was performed on three different transformants carrying the *G. graminis lox* gene (T_1_-T_3_) and three different *G. graminis lox* H306Q-H310E mutant transformants (M_1_-M_3_) for which the results are shown in Figure 3. Western blot analysis revealed *A. nidulans* has two different protein band fingerprints seen in samples WT, T_2_, T_3_, M_2_ and T_1_, M_1_, M_3_ (Figure 3a). This coincides with the single and double band found in the western blot analysis of the intracellular proteins of *A. nidulans* (Figure 3d). The presence of the bands found in the wild type samples showed non-selective binding of polyclonal antibodies. Production of the *G. graminis* LOX was neither detected in the supernatants nor in the cell free extract. Also the mutant version of the *G. graminis* LOX was not be detected in the M_1_-M_3_ samples. Since the expression of the *G. graminis* LOX was not be detected at protein level by SDS-PAGE nor by western blot, a proteomic analysis was performed of the proteins isolated by immunoprecipitation using the polyclonal antibodies against *G. graminis* LOX.

### Comparative proteomics analysis of LOX immuno-precipitated fractions from *A. nidulans* WG505 and *A. nidulans* GG-LOX strains

**Figure 4:**
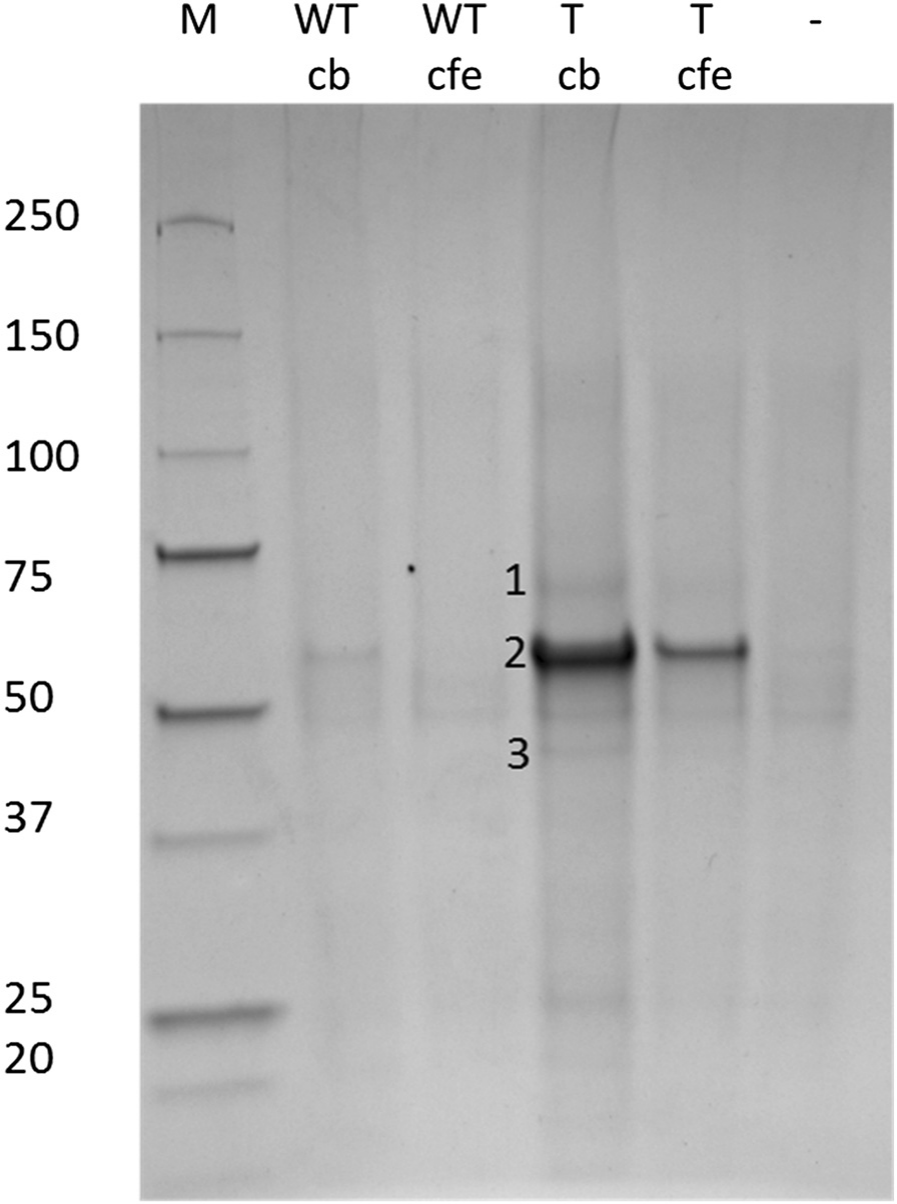
**Immunoprecipitation of*G. graminis*LOX in cell free extract (CFE) and culture broth (CB) of*A. nidulans*wild type (WT) and the transformant (T) using polyclonal antibodies against*G. graminis*LOX.** In sample T_cfe_ a protein band is found at ~67 kDa, which corresponds to the size *G. graminis* LOX using proteomics (1), a protein band of ~54 kDa represents aminopeptidase Y (2), and a protein band ~41 kDa is thioredoxin reductase (3).

Immunoprecipitation was done on both cell free extract and culture broth for the identification of *G. graminis* LOX. Three different proteins were identified in the *A. nidulans* WG505 wild type and the *A. nidulans* carrying the *G. graminis* LOX as shown in Figure 4. Analysis of the *A. nidulans* transformant revealed intracellular production of *G. graminis* LOX (1) but the LOX was not found in the culture broth. Also, aminopeptidase Y (2) was identified in the *A. nidulans* transformant. A third difference is the transformant showed production of thioredoxin reductase (3). This protein functions as a defense agent against oxidative damage and could well be a reflection of the production and activity of *G. graminis* LOX (Missall and Lodge [2005]). Finally a difference is found in the production of the PpoC enzyme. In the *A. nidulans* WG505 wild type this protein is present in the cell free extract and the culture broth. However, only small traces of PpoC were found in the transformant.

### Fermentation of *A. nidulans*

To verify whether the uncontrolled pH, substrate consumption, and oxygen consumption in shake flasks were promoting the degradation of the *G. graminis* LOX a 3 to 5 l fed-batch fermentation was run. Biomass was generated using D-glucose as the carbon source before *A. nidulans* was induced using D-xylose. Samples were taken every 2 h to test for the presence of the *G. graminis* LOX in the medium. However, no *G. graminis* LOX could be detected.

## Discussion

Phenotypic differences between the wild type and the transformant expressing the *G. graminis* LOX were found on both agar plate and in liquid cultures. After 48 h the culture broth of *A. nidulans* GG-LOX changed towards a brown colour. This indicates *A. nidulans* might be stressed due to the production of *G. graminis* LOX. Based on these phenotypic changes and the absence of *G. graminis* LOX activity, it was investigated to study at what stage the LOX production was stopped. By mRNA analysis *G. graminis lox* mRNA could be detected thus, it can be concluded the *lox* gene was successfully transcribed. Therefore the production failed at a different level in the protein synthesis process. Proteins from both strains were isolated and analyzed using *G. graminis* polyclonal antibodies using different methods. Proteomics data revealed production of three proteins in the *A. nidulans* transformant that were not found in the wild type. The presence of significant amounts of protease aminopeptidase Y and the intercellular location of the *G. graminis* LOX suggest the LOX is not secreted but is effectively intracellular degraded. Figure 4 shows *G. graminis* LOX is produced as the 67 kDa version instead of the highly glycosylated one as is found in *G. graminis*. This indicates the *G. graminis* LOX is degraded before it is glycosylated in the secretory pathway. Previous research showed expression of the *G. graminis* LOX in *Pichia pastoris* does not need to be glycosylated to be active and this non-glycosylated LOX could be formed by proteases from the expression host (Cristea et al. [2005]). Also, production of thioredoxin reductase suggests *A. nidulans* responds the oxidative stress caused by the *G. graminis* LOX. Another difference found is the presence of PpoC in the wild type while only traces were found in the transformant. This result suggests the presence and activity of *G. graminis* LOX interferes with the *ppo* oxylipin pathway in *A. nidulans*. The distribution of *ppo* and *lox* genes in *Aspergillus* sp. show *A. nidulans* and *A. niger* have three *ppo* genes and no *lox* genes. *A. fumigatus* contains three *ppo* genes and two *lox* genes, and *A. flavus* contains four *ppo* genes and one *lox* gene (Brown et al. [2008]; Affeldt et al. [2012]; Wadman et al. [2009]; Tsitsigiannis et al. [2005]). This implies the balance of LOX activity in *Aspergillus* is monitored by another oxylipin-producing coding gene. The presence of thioredoxin reductase suggests oxidative stress to the *A. nidulans* GG-LOX transformant and the *G. graminis* LOX is intercellular active. Previous studies showed a concentration of 10–100 μM 9*S*-HPODE and 13*S*-HPODE had effect on mycelial growth (Burow et al. [1997]). Our results show a phenotypic difference between *A. nidulans* wild type and transformant suggesting the oxylipin balance is disturbed by *G. graminis* LOX activity. One might speculate on the repressing activity of *G. graminis* LOX on *ppoC*, since PpoC was only slightly present in the *A. nidulans* transformant. The repressing and up-regulating function of PpoB on *ppoA* and *ppoC* are in line with this hypothesis (Tsitsigiannis et al. [2005]).

Heterologous production of *G. graminis* LOX was successfully performed in *P. pastoris* and *Trichoderma reesei* (Cristea et al. [2005]; Nyyssölä et al. [2012]). A difference between *P. pastoris* and *T. reesei* compared to *Aspergillus* sp. is the presence of these *ppo* genes. The double site-directed H306Q-H310E mutagenesis show that *G. graminis* LOX activity is not causing the low yield of the *G. graminis* LOX. However, the presence of the protease aminopeptidase Y suggests the LOX is degraded. We hypothesize the introduction of the *G. graminis* LOX disturbs the oxylipin balance in *A. nidulans* resulting in a different phenotype and thioredoxin reductase is induced to neutralize the oxidative stress that is caused by the *G. graminis* LOX. Based on the protein composition between *A. nidulans* WG505 and *A. nidulans* GG-LOX, we conclude heterologous production of *G. graminis* LOX using *A. nidulans* as an expression system is not effective for industrial purposes.

## Competing interests

The authors declare that they have no competing interests.

## Authors’ contributions

RH carried out the molecular cloning of *A. nidulans* GG-LOX, mRNA analysis, the western blot experiments, the immunoprecipitation, the proteomics analysis, fermentation experiments, and drafted the manuscript. JPvS was responsible for the mutagenesis work and analysis presented in this paper. JATR conceived and performed the proteomics sample preparation and contributed to the manuscript. RH and LHdG designed the experiments participated to draft the manuscript. All authors read and approved the final manuscript.
